# Effects of adjuvants in a rabies-vectored Ebola virus vaccine on protection from surrogate challenge

**DOI:** 10.1038/s41541-023-00615-z

**Published:** 2023-02-08

**Authors:** Catherine Yankowski, Drishya Kurup, Christoph Wirblich, Matthias J. Schnell

**Affiliations:** 1 Department of Microbiology and Immunology, Sidney Kimmel Medical College at Thomas Jefferson University, Philadelphia, PA USA; 2 Jefferson Vaccine Center, Sidney Kimmel Medical College, Thomas Jefferson University, Philadelphia, PA USA

**Keywords:** Adjuvants, Inactivated vaccines, Ebola virus, Antibodies

## Abstract

Ebola virus is the primary contributor to the global threat of filovirus severe hemorrhagic fever, and Ebola virus disease has a case fatality rate of 50–90%. An inactivated, bivalent filovirus/rabies virus vaccine, FILORAB1, consists of recombinant rabies virus virions expressing the Ebola virus glycoprotein. FILORAB1 is immunogenic and protective from Ebola virus challenge in mice and non-human primates, and protection is enhanced when formulated with toll-like receptor 4 agonist Glucopyranosyl lipid adjuvant (GLA) in a squalene oil-in-water emulsion (SE). Through an adjuvant comparison in mice, we demonstrate that GLA-SE improves FILORAB1 efficacy by activating the innate immune system and shaping a Th1-biased adaptive immune response. GLA-SE adjuvanted mice and those adjuvanted with the SE component are better protected from surrogate challenge, while Th2 alum adjuvanted mice are not. Additionally, the immune response to FILORAB1 is long-lasting, as exhibited by highly-maintained serum antibody titers and long-lived cells in the spleen and bone marrow.

## Introduction

Ebola virus (EBOV) is a member of the *Filoviridae* family and is the primary contributor to the global threat of filovirus severe hemorrhagic fever^1^. FILORAB1 (a portmanteau of filovirus and rabies virus) consists of recombinant rabies virus (RABV) derived from the SAD B19 wildlife vaccine strain and expresses the codon-optimized Ebola virus glycoprotein (EBOV-GP) on its surface. Non-human primates (NHPs) immunized with unadjuvanted BNSP333-GP, the vector containing non-codon-optimized EBOV-GP, had a 50% protection rate from EBOV challenge, and of the protected NHPs, a Th1 antibody response was noted^2^. An additional NHP study found that FILORAB1 formulated with Glucopyranosyl lipid adjuvant (GLA) and a squalene oil-in-water emulsion (SE) was 100% protective from EBOV challenge^3^. GLA activates toll-like receptor 4 (TLR4) signaling and SE activates the NLRP3 inflammasome^4^. These data suggest that GLA-SE improves FILORAB1 immunogenicity and EBOV protection through a Th1 antibody response, but this has not been directly demonstrated.

Many vaccine platforms have been attempted to develop an effective EBOV vaccine^5^. The US Food and Drug Administration (FDA) approved a vesicular stomatitis virus vaccine expressing the Zaire ebolavirus glycoprotein (rVSV-ZEBOV) in 2019^6^. This vaccine has proven to be an effective emergency vaccine to contain EBOV outbreaks and protect laboratory staff and healthcare personnel^7^ but has shortcomings: (1) adverse side-effects have been reported in immunized people including high fever and arthritis; (2) the vaccine requires cold chain storage of −70 °C or lower, an unfeasible requirement for endemic regions; and (3) the vector is replication-competent and therefore a concern in immunocompromised people, young infants, or pregnant women^8,9^. FILORAB1 is heat stable and retains immunogenicity after 2 weeks at 50 °C^10^, and because it is an inactivated vaccine, it is safe for use in immunologically high-risk groups.

The inactivated RABV vaccine is widely used and has a long history of safe administration in humans^11^. Despite the vaccine being 100% effective when administered as a preexposure and, if exposed, postexposure prophylactic, RABV is still responsible for an estimated 59,000 global human deaths annually and is endemic to Africa^12,13^. A RABV-based EBOV vaccine has the advantage of dual protection of vaccinees in coinciding endemic regions. We have extensively studied recombinant RABV vaccines against emerging pathogens^14–20^. A mutation at amino acid position 333 of the RABV glycoprotein attenuates the virus, and administering the vaccine inactivated enhances its safety^19,21,22^. RABV vaccination often protects long-term, and revaccination is rarely needed even after decades of previous immunization. We have shown that the RABV platform elicits long-term immunity to the foreign inserted antigen in a recombinant SARS-CoV-2 vaccine in mice^23^, and FILORAB1 has demonstrated durability of antibody responses in NHPs to 1 year^24^.

The ability of adjuvants to affect vaccine immune responses is an important consideration in vaccine development, especially for inactivated vaccine platforms. Through an adjuvant comparison in mice, we demonstrate that GLA-SE improves FILORAB1 efficacy by activating the innate immune system and shaping a Th1-biased adaptive immune response. We also tested the component adjuvants GLA and SE alone. We included the gold standard and Th2 adjuvant, alum, the aluminum salts used in most FDA-approved vaccines, and a combination GLA-alum. Through a stringent surrogate challenge system utilizing pseudotyped vesicular stomatitis virus, GLA-SE and SE adjuvanted mice are better protected than the Th2 adjuvant alum immunized mice. We also demonstrate the longevity of the immune response to FILORAB1, an important consideration for any vaccine but particularly EBOV, which has had continuous outbreaks for the past several years.

## Results

### FILORAB1 immunization elicits robust but adjuvant-dependent antibody responses

**Fig. 1:**
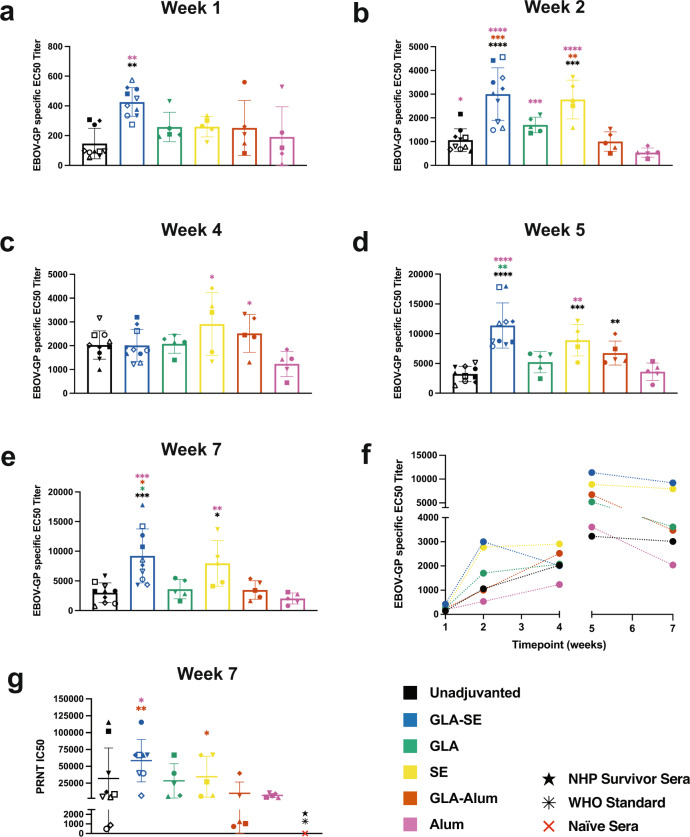
Adjuvant comparison of anti-EBOV-GP serum antibody titers post-immunization with RABV-vectored FILORAB1 vaccine. **a** EBOV-GP specific antibody ELISA of sera from C57BL/6 mice immunized with a prime-boost schedule at day 0 and week 4 with FILORAB1 unadjuvanted or with GLA-SE, SE, GLA-alum, or alum at 1 week, **b** 2 weeks, **c** 4 weeks, **d** 5 weeks, and **e** 7 weeks post-immunization reported as average half-maximal effective concentration (EC50) titer (bars) determined from individual mouse serum (symbols) ELISA curves. **f** EBOV-GP specific antibody responses over time from 1 to 7 weeks post-immunization separated by the boost immunization at week 4. **g** Neutralizing antibody titers at week 7 reported as half-maximal inhibitory concentration (IC50) of serum dilution. NHP survivor sera (black star) is pooled from a previous EBOV challenge experiment. The WHO standard (black asterisk) consists of convalescent plasma pool. Error bars represent the standard deviation (SD) from the mean. Statistics are by one-way ANOVA with post hoc Tukey’s test of log-transformed EC50 or IC50 titers. *p* > 0.1234 (ns), *p* < 0.0332 (*), *p* < 0.0021 (**), *p* < 0.0002 (***), *p* < 0.0001 (****).

C57BL/6 mice were immunized with 10 µg of FILORAB1 formulated with or without adjuvant and analyzed for binding antibody by EBOV-GP ELISA. Along with GLA-SE adjuvanted mice, we included the component adjuvants GLA in an aqueous formulation and SE. Along with the Th2 adjuvant alum (aluminum phosphate), we included a combination adjuvant GLA-alum, which we expected to elicit a more balanced Th1/Th2 response. As early as 1 week post-prime immunization, all mice had detectable EBOV-GP specific binding antibodies by ELISA, but responses were significantly higher in GLA-SE adjuvanted mice (Fig. 1a). This difference increased over time at week 2, with GLA-SE and SE adjuvants having significantly higher titers than unadjuvanted (Fig. 1b). On the day of the boost immunization at week 4, similar titers were seen between groups, with alum mice having the lowest average EC50 titer (Fig. 1c). Peak antibody titers were seen 1 week post-boost immunization at week 5, with GLA-SE, SE, and GLA-alum adjuvanted mice having significantly higher antibody titers than unadjuvanted (Fig. 1d). By week 7, GLA-SE adjuvanted mice had EC50 titers 3 times higher than unadjuvanted (Fig. 1e). Over time, we saw less variance between groups before the boost-immunization, but the boost separated the adjuvants into high-responding and low-responding groups, with GLA-SE and SE adjuvants resulting in antibody titers 3.5 and 2.7-fold higher respectively than unadjuvanted (Fig. 1f). We utilized a replicating recombinant vesicular stomatitis virus (VSV) pseudotyped with EBOV-GP (VSVΔG-EBOV-GP) expressing GFP as a surrogate virus for neutralization assays. By plaque reduction neutralization test (PRNT) of week 7 sera, half-maximal inhibitory concentrations (IC50) for all adjuvant groups were detected but were significantly higher for GLA-SE than GLA-alum (Fig. 1g). We assessed responses to the RABV vector by ELISA for binding antibodies and rapid fluorescent foci inhibition test (RFFIT) for neutralizing antibodies. All mice had RABV-G specific antibodies by ELISA and RFFIT and these were significantly higher for GLA-SE adjuvanted mice (Supplementary Fig. 1a, b).

### Adjuvants highly affect the Th1/Th2 skew of the antibody response

**Fig. 2:**
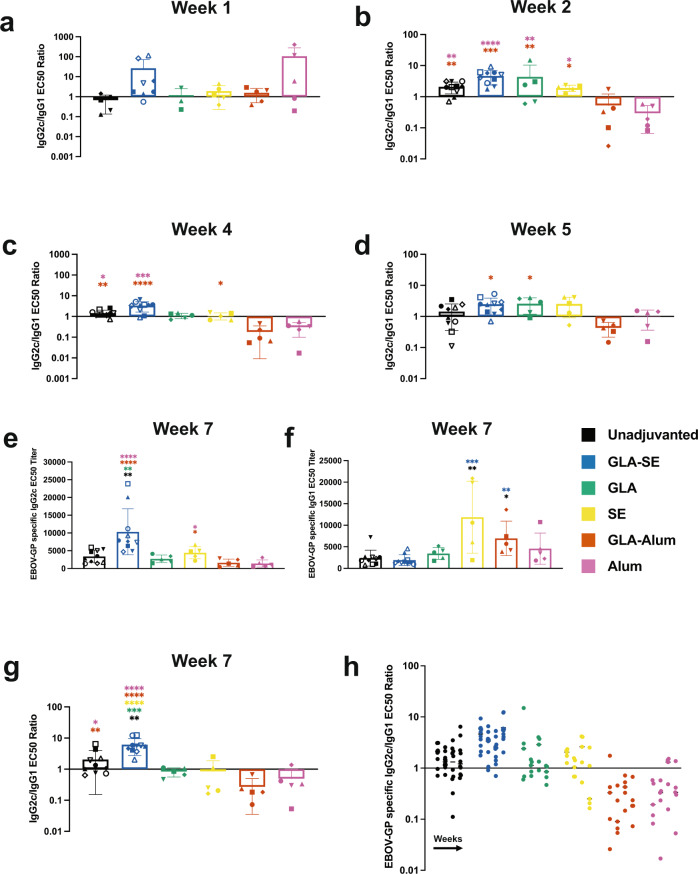
Th1/Th2-associated EBOV-GP specific antibody responses are highly affected by adjuvant formulation. **a** Ratio of IgG2c/IgG1 EBOV-GP specific EC50 titer of sera from mice immunized with FILORAB1 unadjuvanted or with GLA-SE, SE, GLA-alum, or alum at 1 week, **b** 2 weeks, **c** 4 weeks, **d** 5 weeks post-immunization reported as average half-maximal effective concentration (EC50) titer (bars) determined from individual mouse serum (symbols) ELISA curves. **e** EBOV-GP specific IgG2c and **f** IgG1 specific antibody titers at 7 weeks post-immunization. **g** Ratio of IgG2c/IgG1 EBOV-GP specific EC50 titer at week 7. **h** Ratio of IgG2c/IgG1 EBOV-GP specific EC50 titers over time from 2 to 7 weeks post-immunization with each cluster representing an adjuvant group and each column of the cluster a week from 2 to 7. Error bars represent SD from the mean. Statistics are by one-way ANOVA with post hoc Tukey’s test of log-transformed EC50 titers. *p* > 0.1234 (ns), *p* < 0.0332 (*), *p* < 0.0021 (**), *p* < 0.0002 (***), *p* < 0.0001 (****).

To assess the association of helper T cell responses to vaccination, we evaluated the subclass of antibody produced within the IgG isotype, where in C57BL/6 mice, IgG2c is the Th1-associated subclass, and IgG1 is Th2-associated. Then, a ratio of IgG2c to IgG1 EBOV-GP specific EC50 titers (Supplementary Fig. 2) determined bias, where a ratio greater than 1 was considered a Th1-associated response and a ratio less than 1, Th2. The effects of adjuvants on the Th1/Th2 response occurred as early as 1 week post-prime immunization, with varying ratios per group (Fig. 2a). The effect of innate immune activation by adjuvant formulation was more pronounced at week 2. As FILORAB1 is a viral-vectored vaccine, unadjuvanted mice had a slight skew toward the anti-viral Th1 response (Fig. 2b). This skew was significantly increased by the Th1 adjuvant GLA-SE and its component adjuvants. However, the alum adjuvants, including GLA-alum, skewed the anti-viral FILORAB1 immune response toward Th2 with ratios less than 1. This pattern held throughout the experiment both before and after the boost at weeks 4 and 5 (Fig. 2c, d). At week 7, GLA-SE had the highest IgG2c titer (Fig. 2e), and despite overall high antibody titers for the SE adjuvanted group, these were primarily the IgG1 subclass (Fig. 2f). This left only unadjuvanted and GLA-SE mice with Th1-biased antibody responses, with component adjuvants having a more balanced response and alum adjuvants skewing toward Th2 (Fig. 2g). This effect on antibody subclass was rapid and prolonged; with each cluster representing an adjuvant group and each of the 4 columns per adjuvant a week over time (week 2, 4, 5, then 7), only GLA-SE adjuvanted mice maintained a skew toward Th1 from weeks 2–7 (Fig. 2h).

### The adjuvant-dependent adaptive response to FILORAB1 is reflective of innate chemokine and cytokine stimulation

**Fig. 3:**
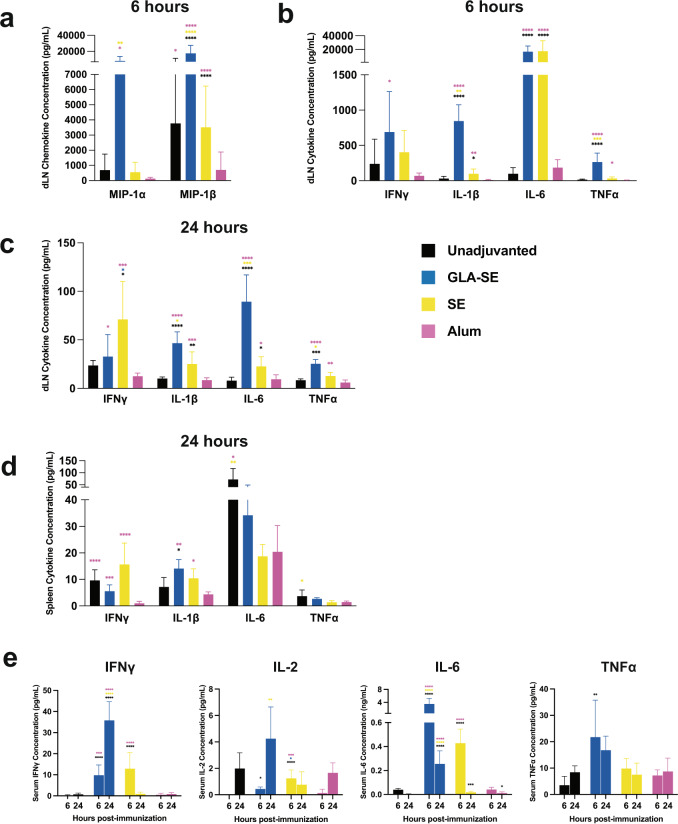
Rapid chemokine and cytokine levels post-immunization are amplified by GLA-SE. **a** Average MIP-1α and MIP-1β levels in pg/mL in C57BL/6 mouse dLNs 6 h post-immunization with FILORAB1 and adjuvant formulation (unadjuvanted, GLA-SE, SE, or alum). **b** IFNγ, IL-1β, IL-6, and TNFα concentrations in dLNs 6 and **c** 24 h post-immunization. **d** IFNγ, IL-1β, IL-6, and TNFα concentrations from immunized mouse spleens. **e** IFNγ, IL-2, IL-6, and TNFα concentrations in serum 6 and 24 h post-immunization. Error bars represent SD from the mean. Statistics are by one-way ANOVA with post-hoc Tukey’s test of log-transformed concentrations. *p* > 0.1234 (ns), *p* < 0.0332 (*), *p* < 0.0021 (**), *p* < 0.0002 (***), *p* < 0.0001 (****).

Having seen such dramatically different EBOV-GP specific antibody titers, we wanted to investigate the effect of these adjuvant groups on the innate immune response to FILORAB1. We immunized mice with a single dose without adjuvant or with GLA-SE, SE, or alum and collected their draining lymph nodes (dLNs), spleens, and serum 6 and 24 h later to assess chemokine and cytokine production. GLA-SE adjuvanted mice had significantly higher concentrations of MIP-1α (CCL3) and MIP-1β (CCL4) in the dLNs 6 h post-immunization (Fig. 3a). These chemokines are responsible for recruiting dendritic cells, monocytes, and inflammatory macrophages to these secondary lymphoid organs. Also at 6 h post-immunization, we saw significantly higher concentrations of IFNγ, IL-1β, IL-6, and TNFα in GLA-SE adjuvanted mice (Fig. 3b). The highest overall cytokine concentrations were for IL-6 in GLA-SE and SE adjuvanted mice, and the amounts were approximately 175 times higher than unadjuvanted. At 24 h post-immunization, cytokine concentrations were lower in the dLNs but highest for GLA-SE and SE adjuvants (Fig. 3c). When culturing splenocytes isolated from mice 24 h post-immunization, SE adjuvanted mice were highest for IFNγ, and all adjuvants were higher than for alum adjuvanted mice (Fig. 3d). IL-1β concentrations from splenocytes were highest in GLA-SE and SE adjuvanted groups, indicative of inflammasome activation. From mouse serum, Th1 associated cytokines IFNγ and IL-2 were highest in GLA-SE adjuvanted mice, with concentrations increasing from 6 to 24 h (Fig. 3e). IL-6 was again highest in GLA-SE and SE adjuvanted mice and peaked at 6 h post-immunization, and TNFα was significantly higher than unadjuvanted in GLA-SE adjuvanted mice at 6 h. Of note, alum adjuvanted mice often had lower responses than other adjuvant groups and unadjuvanted mice (Fig. 3a–e). We completed an expanded screening of chemokine and cytokine concentrations at both 6 and 24 h post-immunization and saw a general decrease over time and overall dramatically higher concentrations in GLA-SE adjuvanted mice (Supplementary Fig. 3).

### GLA-SE and SE adjuvants in FILORAB1 immunization provide total protection from surrogate challenge

**Fig. 4:**
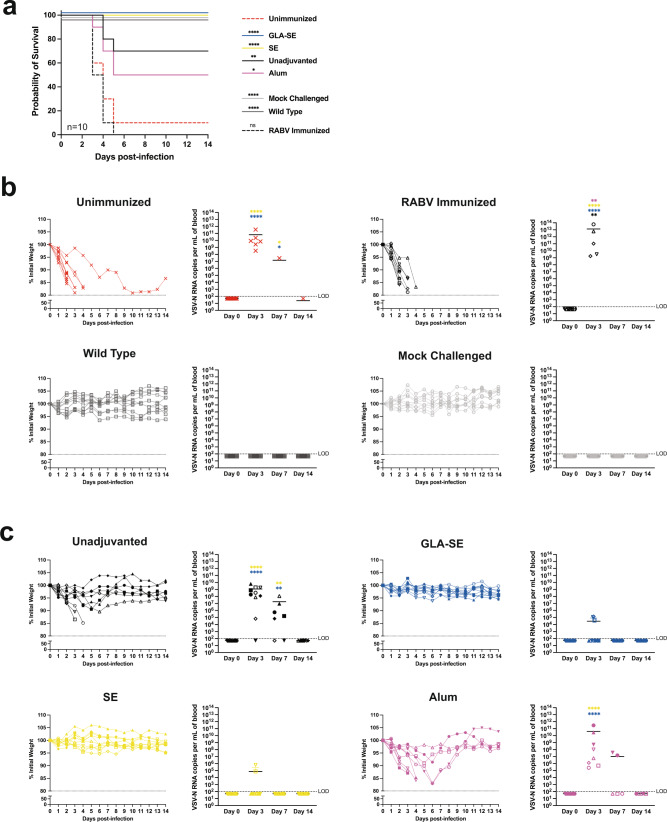
FILORAB1 immunization adjuvanted with GLA-SE or SE provides total protection from surrogate challenge. **a** Survival of *IFNAR*^−/−^ mice challenged intraperitoneally (IP) 4 weeks post-immunization with a lethal dose of VSVΔG-EBOV-GP (5 × 10^5^ PFU). **b** Weight loss and qPCR of VSV-N RNA in experimental control groups including unimmunized, RABV vector immunized, wild type, and mock challenged mice. **c** Weight loss and qPCR of VSV-N RNA in FILORAB1 and adjuvant formulation immunized mice post-challenge. Limit of detection (LOD) is 100 copies per/mL (dotted line). Statistics are by simple survival Kaplan–Meier analysis and one-way ANOVA with post hoc Tukey’s test of log-transformed RNA copy number. *p* > 0.1234 (ns), *p* < 0.0332 (*), *p* < 0.0021 (**), *p* < 0.0002 (***), *p* < 0.0001 (****).

We utilized a recombinant vesicular stomatitis virus pseudotyped with EBOV-GP (VSVΔG-EBOV-GP) as a highly lethal and stringent surrogate challenge model in Interferon α/β receptor deficient (*IFNAR*^−/−^) mice. When infected with VSVΔG-EBOV-GP, naïve mice rapidly lost weight and succumbed to infection within 3–4 days on average, typically within 1 week (Supplementary Fig. 4a, b). Mice that survived to day 3 had viral RNA titers to 10^11^ copies per mL of blood (Supplementary Fig. 4c). We immunized *IFNAR*^−/−^ mice with a single dose of FILORAB1 and various adjuvant groups and challenged after 4 weeks with VSVΔG-EBOV-GP. On the day of challenge, *IFNAR*^−/−^ mice had comparable EBOV-GP specific antibody titers by ELISA to wild-type mice (Supplementary Fig. 4d). Mice also followed the same trend of antibody titers dependent on adjuvant formulation, but, lacking IFNAR signaling, had a more balanced Th1/Th2 response (Supplementary Fig. 5a). After challenge, mice were monitored for survival and weight loss for 14 days. Unimmunized and vector control RABV immunized mice rapidly lost weight and succumbed to infection (Fig. 4a, b). These mice also had high viral RNA titers before they succumbed. Wild type mice had a slight dip in weight after being infected with the high dose of live virus, whereas mock-challenged *IFNAR*^−/−^ mice had no weight loss, and both groups had undetectable viral RNA. GLA-SE and SE adjuvanted mice had 100% protection from challenge with no weight loss and minimal viral RNA detected (Fig. 4c). By contrast, unadjuvanted and alum adjuvanted mice had a 70% and 50% protection rate, respectively, with rapid and prolonged weight loss. These mice also exhibited significantly higher viral RNA titers in the blood than the levels detected in GLA-SE and SE adjuvanted mice. All mice surviving the study cleared the viremia by day 14 post-infection. Antibody titers of surviving mice leveled off between adjuvant groups post-challenge (Supplementary Fig. 5b, c).

### Antibody transfer from FILORAB1 immunized mice is sufficient for protection from surrogate challenge

**Fig. 5:**
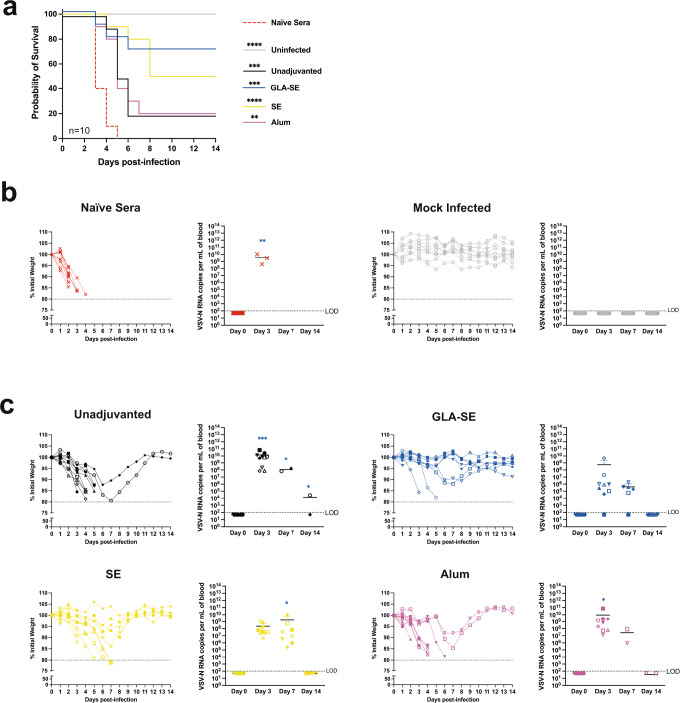
Passive transfer of immunized mouse sera is sufficient for protection from surrogate infection. **a** Survival of *IFNAR*^−/−^ mice infected intraperitoneally (IP) 24 h after passive transfer of C57BL/6 immunized mouse sera. Mice were challenged with a lethal dose of VSVΔG-EBOV-GP (5 × 10^5^ PFU). **b** Weight loss and qPCR of VSV-N RNA in experimental control groups including mice transferred naïve sera and mock challenged mice. **c** Weight loss and qPCR of VSV-N RNA in FILORAB1 and adjuvant formulation immunized mice post-infection. Limit of detection (LOD) is 100 copies per/mL (dotted line). Statistics are by simple survival Kaplan-Meier analysis and one-way ANOVA with post hoc Tukey’s test of log-transformed RNA copy number. *p* > 0.1234 (ns), *p* < 0.0332 (*), *p* < 0.0021 (**), *p* < 0.0002 (***), *p* < 0.0001 (****).

To demonstrate the antibody-mediated role of protection of FILORAB1, we immunized wild type mice and passively transferred sera to *IFNAR*^−/−^ mice. Mice that were transferred naïve sera all succumbed to infection by day 5, whereas GLA-SE adjuvanted mice had a 70% protection rate, and SE mice had a 50% protection rate (Fig. 5a). This differed from unadjuvanted and alum adjuvanted mice that had protection rates of 20%. Mock infected mice maintained weight with undetectable viral RNA in the blood (Fig. 5b). GLA-SE immunized mice had less weight loss compared to other adjuvant groups (Fig. 5c). Mice transferred unadjuvanted, SE, and alum immunized sera had significantly higher viremia than GLA-SE adjuvanted mice post-infection. Of the seven surviving GLA-SE adjuvanted mice, five had viral RNA titers one to threefold lower than the other adjuvant groups, and the other two mice had undetectable viremia, which was evidence for the role of antibody-mediated protection in this system.

### Robust EBOV-GP specific antibody responses are maintained at 1 year in mice

**Fig. 6:**
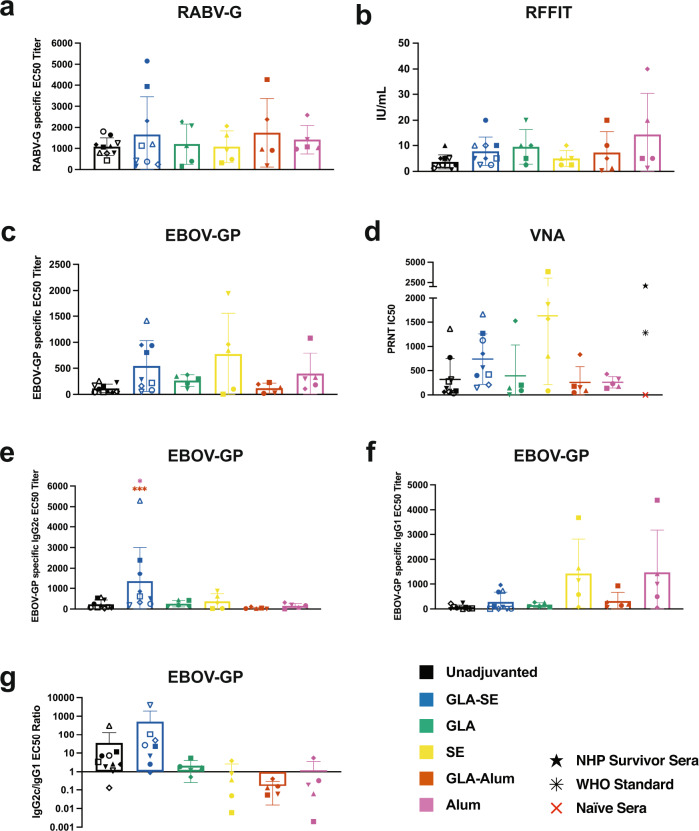
FILORAB1 provides long-term antibody titers 1-year post immunization. **a** RABV-G specific antibody ELISA of sera from C57BL/6 mice immunized with a prime-boost schedule at day 0 and week 4 with FILORAB1 unadjuvanted or with GLA-SE, SE, GLA-alum, or alum at 1 year-post immunization. Data reported as average half-maximal effective concentration (EC50) titer (bars) determined from individual mouse serum (symbols) ELISA curves. **b** RABV-neutralizing antibody titers by RFFIT reported in international units (IU) per mL of average neutralizing titers (bars) of individual mouse serum (symbols). **c** EBOV-GP specific antibody ELISA at 1 year. **d** Neutralizing antibody titers at 1 year reported as half-maximal inhibitory concentration (IC50) of serum dilution. NHP survivor sera (black star) is pooled from a previous EBOV challenge experiment. The WHO standard (black asterisk) consists of convalescent plasma pool. **e** EBOV-GP specific IgG2c, **f** IgG1, and **g** ratio of IgG2c/IgG1 ELISA at 1 year. Error bars represent SD from the mean. Statistics are by one-way ANOVA with post hoc Tukey’s test of log-transformed EC50, IU, or IC50 titers. *p* > 0.1234 (ns), *p* < 0.0332 (*), *p* < 0.0021 (**), *p* < 0.0002 (***), *p* < 0.0001 (****).

Knowing that RABV vaccination results in long-term immunity and having prior evidence that this long-term property is conferred to foreign antigens in the recombinant RABV vaccine platform, we tested antibody titers 1 year post-FILORAB1 immunization in mice. As our vector control, we observed RABV-G antibody titers in all adjuvant groups with nonsignificant differences in antibody subclass ratio (Fig. 6a and Supplementary Fig. 6). By RFFIT, we also determined RABV neutralizing titers above the accepted protective level of 0.5 IU/mL except for one mouse from the GLA-alum group (Fig. 6b). EBOV-GP antibody titers were highly maintained as well, with GLA-SE, SE, and alum groups the highest and with neutralizing titers in all groups (Fig. 6c, d). However, the SE and alum groups primarily maintained the IgG1 subclass, where GLA-SE maintained IgG2c (Fig. 6e, f). This was demonstrated by the IgG2c/IgG1 ratios, where GLA-SE mice showed the greatest skew toward Th1 after 1 year, and the alum adjuvants along with SE showed a long-term skew toward Th2 (Fig. 6g).

### FILORAB1 immunization elicits long-lived antibody-secreting cells

**Fig. 7:**
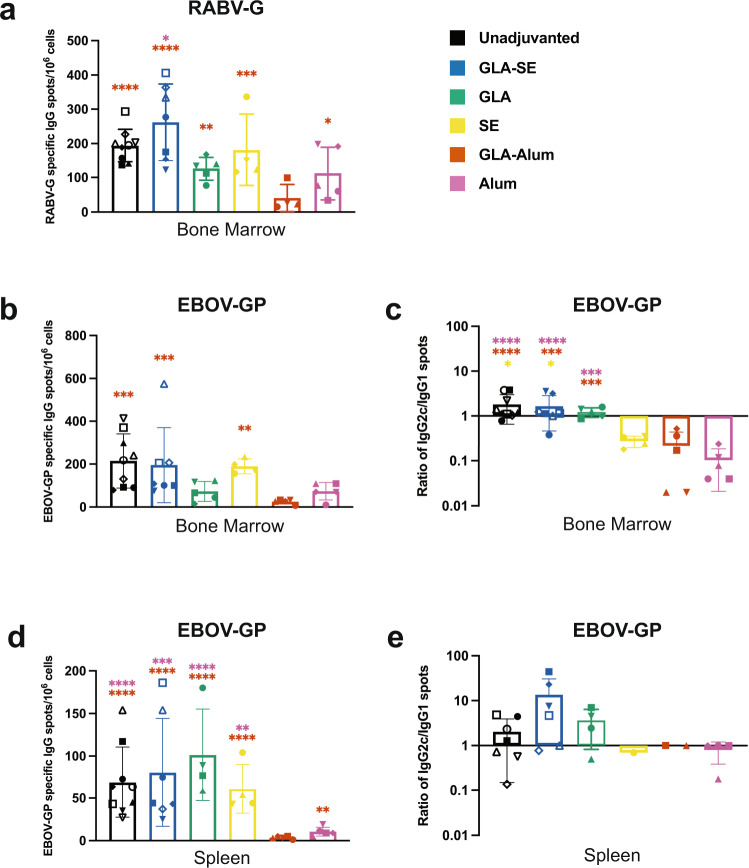
Long-term antibody responses to FILORAB1 are maintained by long-lived antibody-secreting cells (ASCs) in the bone marrow and spleen. **a** The average number (line) of RABV-G and **b** EBOV-GP specific ASCs present in individual mouse bone marrow (symbols) at 1 year per adjuvant group. **c** The ratio of IgG2c/IgG1 ASCs specific to EBOV-GP in the bone marrow. **d** The average number of EBOV-GP specific ASCs present in individual mouse spleens at 1 year per adjuvant group. **e** The ratio of IgG2c/IgG1 ASCs specific to EBOV-GP in the spleens. Statistics are by one-way ANOVA with post hoc Tukey’s test of the log-transformed total number of cells or ratios. *p* > 0.1234 (ns), *p* < 0.0332 (*), *p* < 0.0021 (**), *p* < 0.0002 (***), *p* < 0.0001 (****).

At 1 year post-immunization, we also investigated the presence of antibody-secreting cells in the spleen and bone marrow of these mice. We detected many RABV-G-specific long-lived cells in the bone marrow (Fig. 7a) and many for EBOV-GP (Fig. 7b). The subclass that these cells were secreting was dependent on the adjuvant at the time of immunization, where unadjuvanted, GLA-SE, and GLA adjuvanted mice primarily had long-lived cells in the bone marrow secreting IgG2c and SE, GLA-alum, and alum mice had cells secreting more IgG1 (Fig. 7c and Supplementary Fig. 7a, b). We observed similar responses in the spleen, with all adjuvant groups more abundant for EBOV-GP antibody-secreting cells than GLA-alum adjuvanted mice (Fig. 7d). The subclass specificity of these cells followed the same pattern as the bone marrow, with GLA-SE adjuvanted mice showing the greatest skew toward Th1 in the spleen (Fig. 7f and Supplementary Fig. 7c, d). We believe these cells were the primary contributor to the highly maintained anti-EBOV-GP serum antibody titers in our long-term experiments.

## Discussion

We have demonstrated the effects of adjuvant selection on immunogenicity of a rabies-vectored Ebola virus vaccine, FILORAB1. FILORAB1 has proven successful in both mouse and NHP studies, enhanced by the Th1 adjuvant GLA-SE^2,15,25–27^. We hypothesized that FILORAB1 immunization produces a Th1-biased antibody response, and that vaccine efficacy is increased by GLA-SE. Here, we tested the component adjuvants GLA and SE and the Th2 adjuvants GLA-alum and alum. We demonstrated that FILORAB1 and GLA-SE immunization resulted in the highest antibody titers and Th1-bias compared to other adjuvant groups. GLA-SE and SE adjuvants rapidly activated the innate immune response. We observed high concentrations of IL-6 in dLNs of these mice, indicative of T and B cell activation and proliferation^28,29^, contributing to the more robust antibody titers in these groups.

A limitation of our study is the use of C57BL/6 mice that are naturally Th1-biased. Additional strains and an outbred model would be beneficial to recapitulate our results. However, alum was able to skew the FILORAB1 immune response to Th2 in C57BL/6 mice, allowing us to test our hypothesis through surrogate challenge with VSV-ΔG-EBOV-GP in *IFNAR*^−/−^ mice. Alum adjuvanted mice had suppressed immune responses and limited protection from surrogate challenge, whereas GLA-SE and SE adjuvanted mice were protected. In vitro, we evaluated the neutralizing function of these antibodies by PRNT, as described previously^30^. In vivo, by passive transfer of serum, protection was shown to be antibody-mediated. However, protection was dependent on the antibody titers induced by the adjuvant, with GLA-SE and SE mice better protected than alum and unadjuvanted. The surrogate challenge system also demonstrated that FILORAB1 is effective in immunocompromised *IFNAR*^−/−^ mice.

NHPs remain the best animal model of Ebola virus disease (EVD)^31,32^. Immunocompetent mice are resistant to EBOV and mouse-adapted (MA)-EBOV has been generated by serial passage^33,34^. In a previous study, an earlier generation of FILORAB1 without codon-optimized GP incorporation and without adjuvant was 100% protective against MA-EBOV in BALB/c mice^15^ but only partially protective in NHPs^3^. These results indicate that MA-EBOV may not be stringent enough for preclinical studies but can still provide valuable results for the design of NHP studies. VSV-ΔG-EBOV-GP (VSV-EBOV) in *IFNAR*^−/−^ mice is an advantageous surrogate for evaluating vaccine response in non-biosafety level 4 (BSL-4) facilities. VSV-EBOV infection studies in rodents showed that F344/N rats and BALB/c mice were unaffected, but Syrian hamsters succumbed within 4 days^35^. However, VSV-EBOV only contains the GP of EBOV and does not fully recapitulate EVD. Since VSV-EBOV is fast-replicating and highly lethal in *IFNAR*^−/−^ mice, it can serve as an appropriate initial challenge model in non-BSL-4 settings. VSV-EBOV only allowed determination of survival, weight loss, and viral RNA copies in the blood. Other rodent models can monitor viral load in the liver, spleen, and other tissues^36^, as well as kidney dysfunction and liver damage that correlate with EVD in humans^37,38^. In this study, VSV-EBOV allowed us to screen several adjuvants and compare their protective efficacy through a surrogate challenge system in a BSL-2 setting. Such experiments in NHPs would require a large number of animals, and therefore the results presented here serve as an intermediate step.

Our results highlight that FILORAB1 maintains long-term antibody titers by ELISA, VNA, and ELISpot detection of antibody-secreting cells in the spleen and bone marrow. The RABV vector system conferred long-term immunity to EBOV-GP specific antibody titers maintained up to 1 year. These GP specific antibodies also recapitulated the isotype subclass of the original induction post-immunization. Although we have demonstrated protection from surrogate challenge, we have not demonstrated protection after an extended time post-vaccination. Pre-challenge *IFNAR*^-/-^ mice that were protected from challenge after a single dose have a similar range of IC50 titers as wild-type mice 1 year post-immunization. The longevity of a protective antibody response needs to be directly demonstrated and may implicate the role of immune memory, which has not been investigated.

Many vaccine strategies have been attempted for an effective EBOV vaccine including replicating and non-replicating vectors, subunit vaccines, DNA, virus-like particles (VLPs), and nanoparticles^39–41^. Immunogenicity studies of these platforms begin in mouse models. Chimpanzee adenovirus type 7 with a heterologous boost protected mice long-term^42^, as did rVSV-ZEBOV in which mice maintained high GP-specific IgG^43^. Adjuvant studies in mice have involved the VLP vaccine strategy^44–48^, including long-term protection by VLPs adjuvanted with GLA-SE^49^. Prompted by the 2014–2016 West African outbreak, research in NHPs and clinical trials was accelerated^50^, and several platforms have displayed longevity in NHPs^24,51,52^. An adjuvanted VLP vaccine that was protective in mice^53^ also protected NHPs^54^. Adjuvanted subunit vaccine strategies have also demonstrated efficacy^55,56^. A subunit nanoparticle vaccine adjuvanted with Matrix-M protected mice^57^ and, in a later report, was effective in humans^58^. The findings of the present study with FILORAB1 and GLA-SE are significant because the formulation has predictive efficacy in NHPs^24,27^.

By network meta-analysis of all EBOV vaccines in humans, FDA-approved rVSV-ZEBOV exhibited the most risk-benefit followed by the rAd5.ZEBOV-GP and ChAd3-EBO-Z vaccines^59^. Ring-vaccination of rVSV-ZEBOV was exceptionally useful in outbreak settings^7^, but long-term responses in humans showed an overall decline in EBOV-GP specific antibodies at 1 year coupled with lower seroconversion rates^60^. Neutralizing antibody titers had seropositivity dropping to 27–31% at 6 months but were overall promising; durability was observed at 2 years, the longest demonstrated for an EBOV vaccine^61,62^. The rVSV-ZEBOV vaccine requires storage at −70 °C or less and has limited viability at higher temperatures^8^. However, FILORAB1 maintains antigenic stability and immunogenicity when stored at 37 °C or higher^63^. Additionally, rVSV-ZEBOV is not suitable for pregnant women, when the risk of EVD is more severe^64^. The inactivated RABV vaccine has been safely administered during pregnancy, suggesting FILORAB1 could be used in this vulnerable population^65^.

The GLA-SE adjuvant was developed by the Infectious Disease Research Institute (IDRI) and has been extensively characterized^66–70^ and used in clinical trials of protein subunit vaccines^25,71–74^. The adjuvant induces a potent Th1 immune response by engaging both TLR4 and the NLRP3 inflammasome pathways^4^. To the best of our knowledge, this is the first demonstration that GLA-SE is highly effective in a viral-vectored vaccine where the FILORAB1 antigen (180 nm) is larger than GLA-SE (100 nm). It is yet to be determined if GLA-SE and FILORAB1 are simultaneously endocytosed by the same antigen-presenting cell or whether another mechanism induces the immune response. Alum is the most widely-used vaccine adjuvant^75^ but dampens the FILORAB1 immune response, as shown by EBOV-GP specific antibody titers, innate immune activation, and long-term responses. This result implies that vaccines requiring a Th1 response to protect against viral pathogens are inhibited by alum. Alum, monophosphoryl lipid A (MPLA), squalene, CpG, and QS-21 are among the adjuvants used in FDA-approved vaccines^75,76^, and this study further highlights the importance of new adjuvant discovery and investigation.

The results reported here demonstrate the influence of adjuvants on the efficacy of inactivated RABV-based EBOV vaccine FILORAB1. Unadjuvanted, the vaccine induced a modest Th1 response in Th1-baised C57BL/6 mice that could be significantly augmented by GLA-SE or biased toward Th2 by alum. GLA-SE and SE adjuvants induced potent innate immune cell activation that resulted in significantly higher antibody titers and protection from surrogate challenge. Serum antibodies demonstrated neutralizing activity in vitro and provided protection by passive transfer in vivo. In long-term studies, antibody titers were maintained together with adjuvant-dependent bias toward Th1 or Th2. These results highlight the importance of adjuvant selection for improving vaccine design and provide a basis for further studying the mechanism of protection of FILORAB1.

## Methods

### Vaccines

The recombinant rabies vaccines BNSP333 and FILORAB1 were constructed, recovered, purified with sucrose or high-pressure concentration, inactivated with β-propiolactone (BPL), and characterized. Briefly, the virus was used to inoculate Vero cells seeded in Cellstack Culture Chambers (Corning) and propagated in VP-SFM medium (Thermo Fisher Scientific) over a period of 18 days. Supernatant collected on day 10 post-infection was filtered through 0.45 μm PES membrane filters (Nalgene). For BNSP333, supernatant was layered onto 20% sucrose in DPBS and virions were sedimented by ultracentrifugation in an SW32 rotor for 1.5 h at 100,000×*g*. Viral particles were resuspended in phosphate-buffered saline (PBS). For FILORAB1, supernatant underwent high-pressure concentration. Both vaccines were inactivated with 50 μL per 1 mg of particles in a 1:100 dilution of BPL (Millipore Sigma, Cat# P5648) in cold water. The absence of infectious particles was verified by inoculating BSR cells with 10 μg of BPL-inactivated viruses over three passages. The vaccines were stored at −80 °C before use.

### Animal ethics statement

This study was carried out in strict adherence to recommendations described in the Guide for the Care and Use of Laboratory Animals, the Office of Animal Welfare, and the United States Department of Agriculture. All animal work was approved by the Institutional Animal Care and Use Committee (IACUC) at Thomas Jefferson University. All procedures were carried out under isoflurane anesthesia by trained personnel and under the supervision of veterinary staff. Mice were housed in cages in groups of five, under controlled humidity and temperature conditions, and 12-h light and dark cycles. Food and water were available ad libidum.

### Immunizations

Groups of five 6–8 week old C57BL/6 mice were purchased from Jackson Laboratories. GLA-SE adjuvanted mice were immunized with 5 µg GLA in 10% SE (IDRI). GLA mice were immunized with 5 µg GLA in an aqueous formulation, and SE mice were immunized with 10% SE in a 2% final concentration (IDRI). Alum adjuvanted mice were immunized with 100 µg Adju-Phos (alum, Invivogen), and GLA-Alum mice were immunized with an additional 5 µg of GLA. 10 µg of chemically inactivated FILORAB1 particles were mixed by pippetting with adjuvant and Tris-Arginine buffer to 100 µL total volume per mouse. Mice were inoculated intramuscularly with 50 µL of the formulation in each hind leg caudal thigh and boosted with the same amount of virus 4 weeks later. Blood samples were collected by retro-orbital bleed before the first immunization until the study endpoint.

### Antigen production for ELISA and ELISpot

For the production of HA-tagged EBOV-GP, sub-confluent T175 flasks of 293 T cells (human kidney cell line) were transfected with a eukaryotic expression vector (pDisplay) that expresses HA-tagged EBOV-GP and purified over a column. Fractions were collected and analyzed by Western Blot, and peak fractions were pooled and dialyzed against PBS in 10,000 molecular weight cutoff dialysis cassettes (Thermo Fisher Scientific) to remove excess HA peptide. After dialysis, the protein was quantitated by UV spectrophotometry and frozen in small aliquots at −80 °C. Stripped RABV Glycoprotein (G) antigen was produced by infecting BEAS-2B cells with rVSV-ΔG-RABV-G-GFP in OptiPRO SFM. Viral supernatants were concentrated and ultracentrifuged through a 20% sucrose cushion. Viral pellets were then resuspended in detergent-containing buffer and centrifuged to strip antigen from the virus. All antigens were further analyzed and characterized by SDS-PAGE gel and Western Blot.

### anti-EBOV-GP and anti-RABV-G IgG ELISA

Immulon 4 HBX 96-well flat-bottom Microtiter plates were coated overnight at 4 °C with 50 ng/well of recombinant EBOV-GP or RABV-G diluted in 15 mM Na_2_CO_3_, 35 mM NaHCO_3_ coating buffer. The plates were washed three times with 300 μL of PBS containing 0.05% Tween-20 (PBST), as were all succeeding washes, and then blocked for 2 hr at room temperature (RT) in 5% Milk in PBST. The plates were washed, and mouse sera were added at a 1:50 starting dilution and further diluted 3-fold down the plates. Plates were kept at 4 °C overnight, washed, and incubated for 2 hr at RT with 100 μL per well horseradish peroxidase-conjugated goat anti-mouse IgG (H + L) (Jackson ImmunoResearch, Cat# 115-035-003), IgG-Fc (Cat# 115-005-008), IgG2c (Cat# 115-035-208), or IgG1 (Cat# 115-035-205) antibody diluted in PBST 1:20,000. The plates were then washed and developed by the addition of 200 μL per well of o-Phenylenediamine Dihydrochloride substrate. The reaction was stopped after 15 min by adding 50 μL per well of 3 M sulfuric acid. The plates were read at the absorbance wavelength of 630 nm (background) and 490 nm (experimental) on a BioTek ELx800 Plate Reader with Gen5 software. The 630 nm reading was subtracted from the 490 nm reading to calculate the delta value analyzed in GraphPad Prism 9 software.

### Surrogate virus production

VSV-ΔG-EBOV-GP was grown and titered on Vero CCL81 cells. Specifically, Vero cells were cultured with VP-SFM supplemented with 1% Pen–Strep, 2× GlutaMAX™, and 10 mM HEPES buffer and infected with a multiplicity of infection (MOI) of 0.01. Virus was harvested when an 80% cytopathic effect was detected and titered by plaque forming assay using 2% methyl cellulose overlay.

### Plaque reduction assay

Anti-EBOV neutralizing antibodies were analyzed from immunized mouse sera heat inactivated for 30 min at 56 °C. 2-fold dilutions of sera were mixed with 100 plaque forming units (PFU) of VSV-ΔG-EBOV-GP expressing GFP and incubated at 34 °C for 1 h in the presence of 5% guinea pig serum complement. A plaque assay was performed in a 96-well plate with Vero CCL81 cells overlayed with 2% methyl cellulose in DMEM. The percentage of plaque reduction was calculated using the GFP signal to determine infection, followed by comparing the number of PFU in the neutralized sample to the input virus. PRNT IC50s were calculated by log(inhibitor) vs normalized response with variable slope in GraphPad Prism 9 software.

### Luminex assay of innate immune chemokines and cytokines

Groups of five 6–8 week old female C57BL/6 mice were immunized intramuscularly as described above with a single dose of FILORAB1 and sacrificed 6 or 24 h later, collecting blood by cardiac puncture. Spleens and draining poplietal and inguinal lymph nodes were harvested in PBS containing 1× HALT protease inhibitor and homogenized. For the spleens, 1 × 10^7^ cells were cultured in DMEM in a 24-well plate for 24 h. Supernatants were analyzed for cytokine and chemokine concentration by Luminex magnetic bead panel (Milliplex) and analyzed on a MAGPIX instrument.

### Surrogate virus challenge

Groups of five 6–8 week old male and female *IFNAR*^−/−^ mice were immunized intramuscularly as described above with a single dose of FILORAB1 or RABV vaccine BNSP333. Immunized *IFNAR*^−/−^ mice were challenged 4 weeks later intraperitoneally (IP) with 5 × 10^5^ PFU in 100 µL of PBS. Mice were monitored daily and sacrificed if weight loss reached 20% or if severe clinical signs of disease were observed. Mice were bled at days 0, 7, and 14 for EBOV-GP specific ELISA and VSV-N qPCR and additionally at day 3 for VSV-N qPCR. For passive transfer experiments, groups of five 6–8 week old female C57BL/6 mice were immunized intramuscularly as described above with a two-dose regimen of FILORAB1 and sacrificed 1-week post boost by cardiac puncture. 300 µL of pooled sera was transferred to *IFNAR*^−/−^ mice, followed by the same infection and post-infeciton monitoring as described above.

### RNA extraction and quantitative real-time polymerase chain reaction (RT-qPCR)

50 µL of whole blood was added to 300 µL of TRIzol LS Reagent and 50 µL of DPEC water. The protocol for RNA extraction of biological fluids with TRIzol LS Reagent was used up to the phase separation step, followed by the PureLink RNA Mini Kit (Ambion). For RT-qPCR, VSV-N RNA was prepared as a standard, and the qPCR followed the protocol for TaqMan Fast Virus 1 Step Master Mix reagent (ThermoFisher), using 5 µL of RNA per reaction with a 60 °C annealing temperature.

### Rabies virus neutralization by RFFIT

Rapid fluorescent focus inhibition tests (RFFITs) were performed as previously described in 1973 by Smith et al. Briefly, mouse neuroblastoma cells (NA) cultured in serum-enriched RPMI media were seeded in 96-well plates and incubated for 48 hr. Independently, individual mouse serum was serially diluted threefold with a starting dilution range of 1:10 to 1:400, depending on the time point of the sera collection. A pre-diluted mixture of RABV strain CVS-11, previously determined to achieve 90% infection in confluent NA cells, was added to each serum dilution. Along with the sera dilutions, the U.S. standard rabies immune globulin (WHO STD) at a starting dilution of 2 international units (IU) per mL was incubated with the virus mixture for 1 h at 34 °C. The medium was then removed from the NA cell plate, replaced with the sera/virus mix, and incubated for 2 h at 34 °C. Post infection, the sera/virus mixture was aspirated and replaced with fresh medium. The plates were then incubated for 22 h (24-h total infection) at 34 °C. After incubation, cells were fixed with 80% acetone, dried, and stained with FITC anti-RABV N Monoclonal Globulin overnight at 34 °C. Wells were assessed for percent infection using a fluorescent microscope. The Reed-Muench method was used to calculate 50% endpoint titers (EPT); these were converted to IU/mL by comparing them to that of the WHO STD.

### Enzyme-linked immunosorbant spot (ELISpot) assay

An ELISpot assay was used to quantify the number of EBOV-GP and RABV-G specific antibody-secreting cells in mouse bone marrow and spleen samples. ELISpot plates were coated with 10 μg/mL EBOV-GP or RABV-G in PBS overnight at 4 °C. Plates were washed with PBS and blocked with goat serum for 1 h at 37 °C followed by 4 °C during the preparation of cells. Bone marrow was harvested from the mouse femurs and tibias. These and the spleens were homogenized followed by red blood cell lysis in ACK buffer; 1.5 × 10^6^ cells were added to antigen-coated plates, serially diluted, and incubated at 37 °C for 16 h. Plates were washed with PBST and incubated in HRP-conjugated goat anti-mouse IgG (H + L) (Jackson ImmunoResearch, Cat# 115-035-003), IgG2c (Cat# 115-035-208), or IgG1 (Cat# 115-035-205) diluted 1:1600 in PBST for 1–2 h at 37 °C. Plates were washed with PBST, followed by PBS, and the TrueBlue peroxidase substrate was added. The reaction was quenched with water, and plates were counted on an AID ELISpot reader.

### Statistical analysis

For the ELISA, log-transformed half-maximal effective concentration (EC50) titers were determined by a threefold dilution series of delta OD value (OD 490–630 nm). For all statistical analyses, one-way ANOVA with post hoc Tukey’s test was performed on log-transformed data for each time point. *p* > 0.1234 (ns), *p* < 0.0332 (*), *p* < 0.0021 (**), *p* < 0.0002 (***), *p* < 0.0001 (****).

### Reporting summary

Further information on research design is available in the Nature Research Reporting Summary linked to this article.

## Supplementary information

Supplementary Material

REPORTING SUMMARY

## Data Availability

All relevant data are available from the corresponding authors upon request.
