# Supplementary material for: Effects of adjuvants in a rabies-vectored Ebola virus vaccine on protection from surrogate challenge
Source: NPJ Vaccines. 2023 Feb 8;8:10. doi: 10.1038/s41541-023-00615-z (PMC9906604; doi:10.1038/s41541-023-00615-z)
Supplement: Supplementary file 1 — Supplementary Material [file 41541_2023_615_MOESM1_ESM.pdf]

Supplementary Figure 1

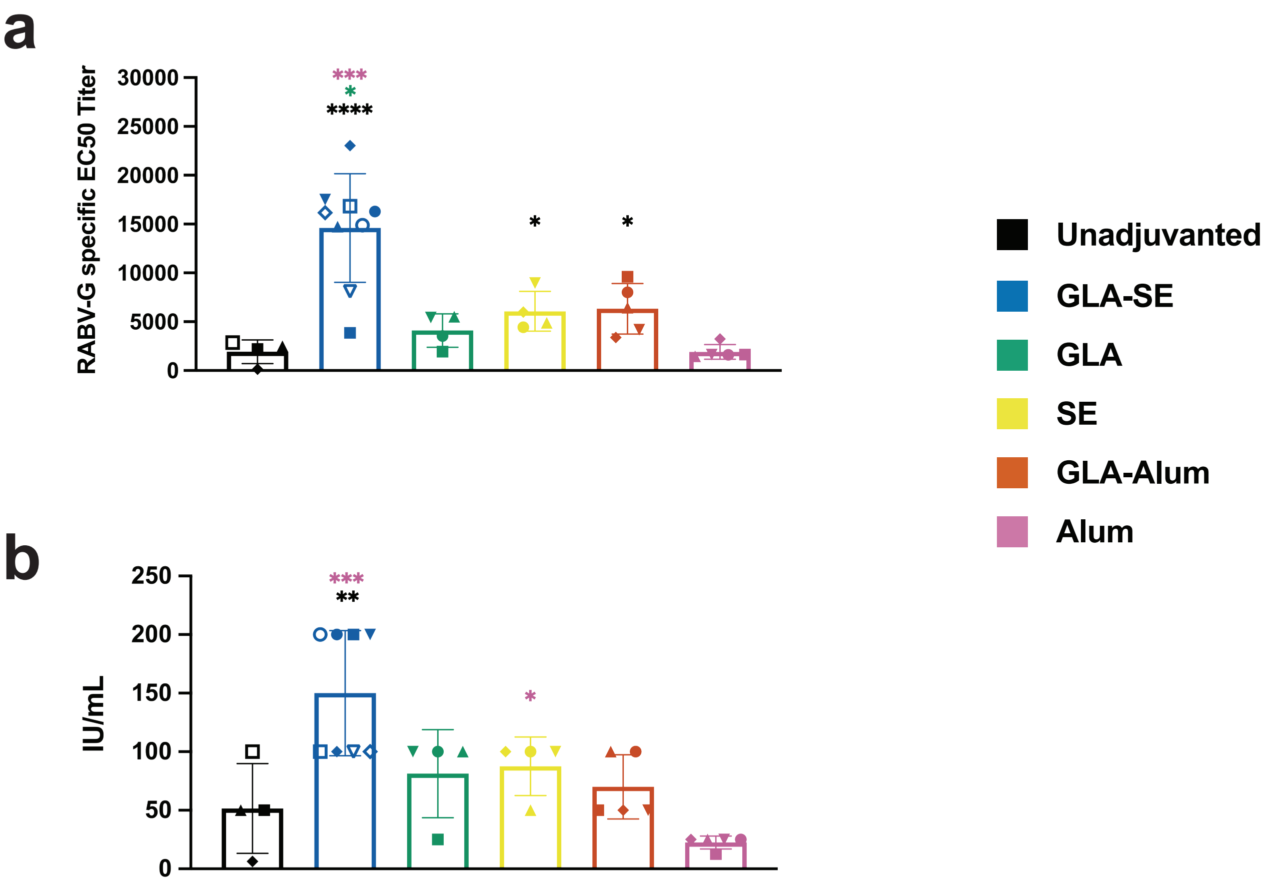

Supplementary Figure 1. Adjuvant comparison of antibody response to RABV vector. **(a)** RABV-G specific antibody ELISA of sera from C57BL/6 mice immunized with a prime-boost schedule at day 0 and week 4 with FILORAB1 unadjuvanted or with GLA-SE, SE, GLA-alum, or alum at 7 weeks post-immunization. Data reported as average half-maximal effective concentration (EC50) titer (bars) determined from individual mouse serum (symbols) ELISA curves. **(b)** RABV-neutralizing antibody titers by RFFIT reported in international units (IU) per mL of average neutralizing titers (bars) of individual mouse serum (symbols). Error bars represent SD from the mean. Statistics are by one-way ANOVA with post-hoc Tukey's test of log-transformed EC50 or IU titers. $p > 0.1234$ (ns), $p < 0.0332$ (*), $p < 0.0021$ (**), $p < 0.0002$ (***), $p < 0.0001$ (****).

Supplementary Figure 2

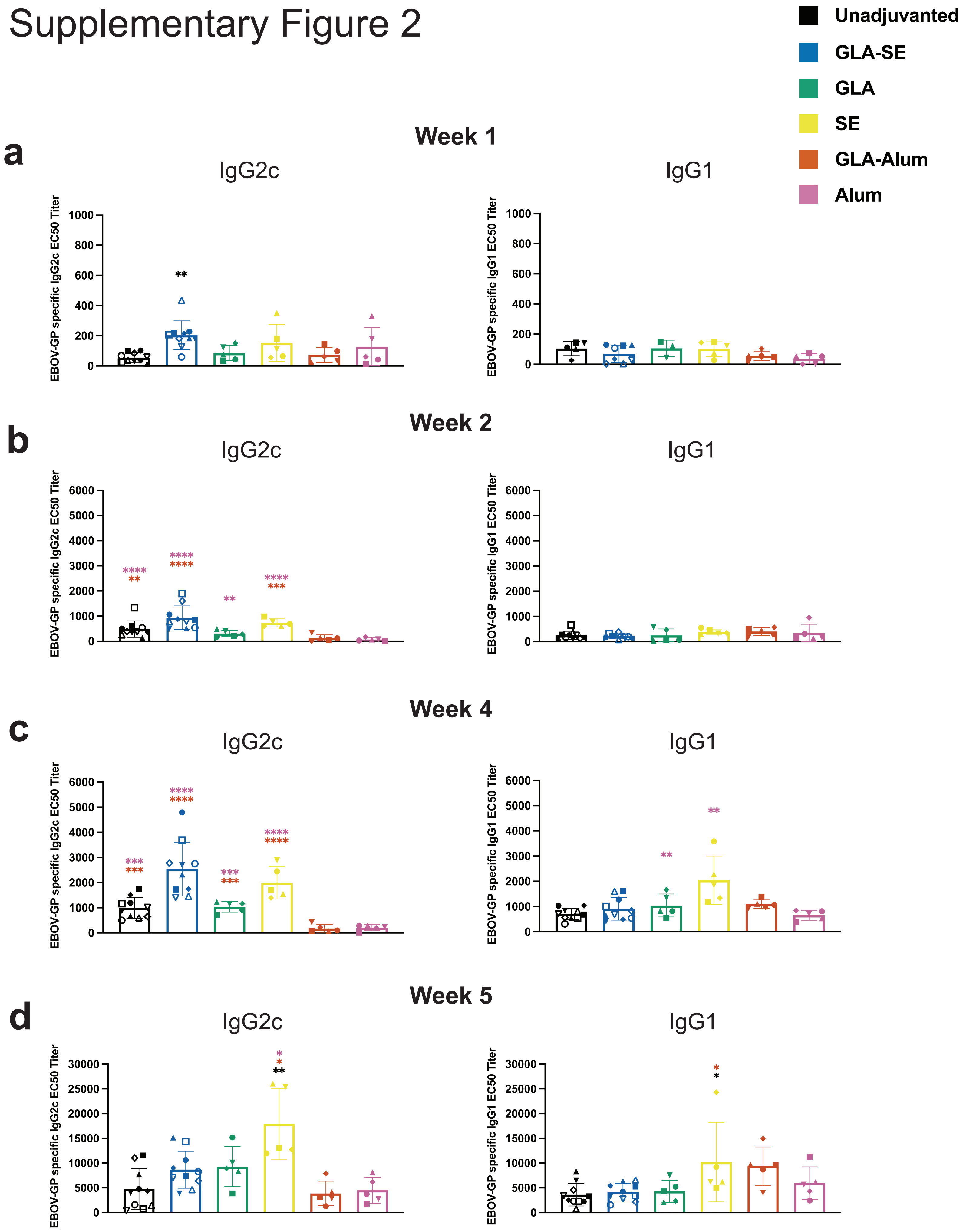

Supplementary Figure 2. Adjuvant comparison of EBOV-GP specific isotype subclass responses. **(a)** EBOV-GP specific IgG2c (left) and IgG1 (right) EC50 titers of C57BL/6 mice at week 1, **(b)** week 2, **(c)** week 4, and **(d)** week 5. Data reported as average half-maximal effective concentration (EC50) titer (bars) determined from individual mouse serum (symbols) ELISA curves. Error bars represent SD from the mean. Statistics are by one-way ANOVA with post-hoc Tukey's test of log-transformed EC50 titers. $p > 0.1234$ (ns), $p < 0.0332$ (*), $p < 0.0021$ (**), $p < 0.0002$ (***), $p < 0.0001$ (****).

Supplementary Figure 3

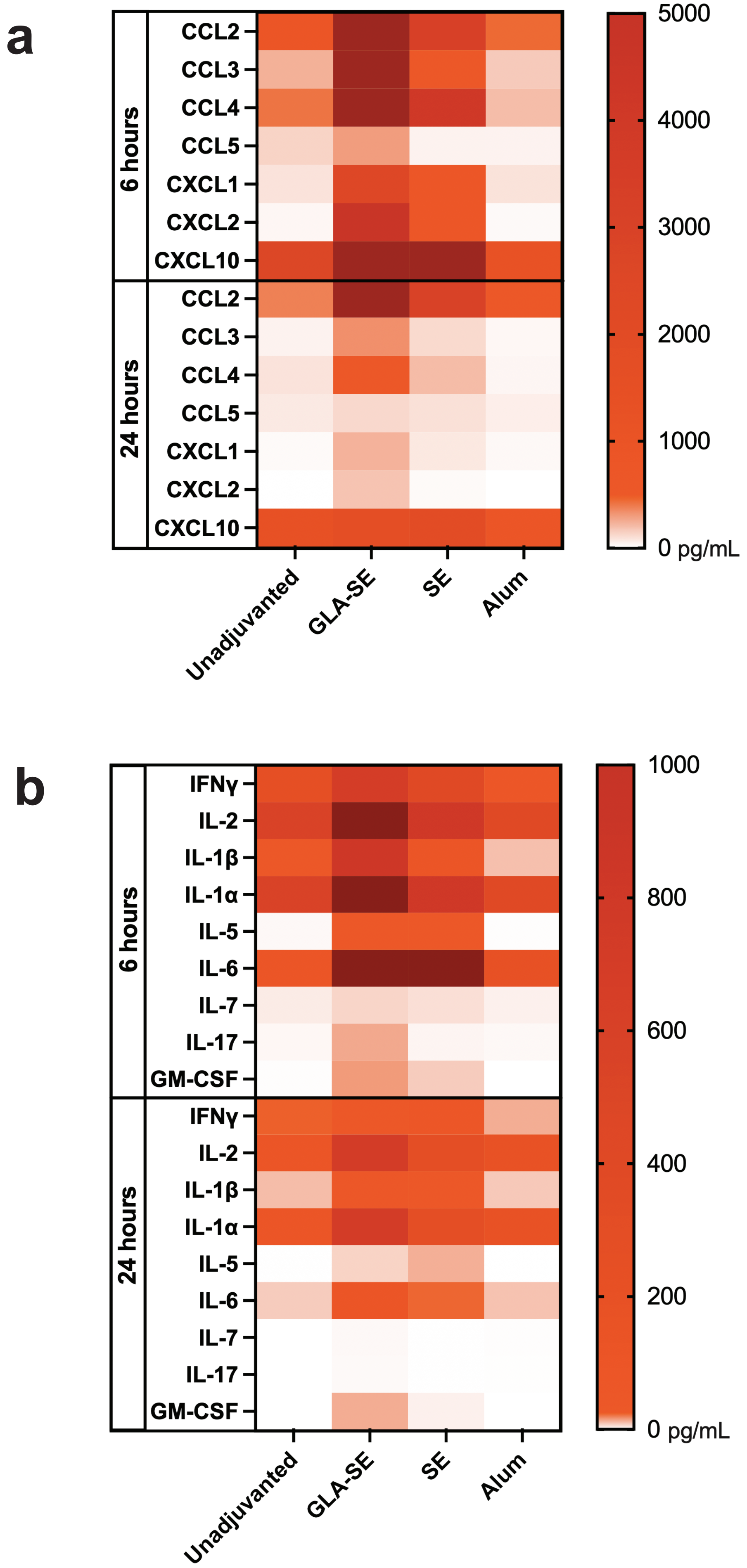

Supplementary Figure 3. Expanded panel of cytokine and chemokine levels in the draining lymph nodes (dLNs). **(a)** Chemokine and **(b)** cytokine concentrations at 6 and 24 hours post-immunization represented by heat map in pg/mL.

Supplementary Figure 4

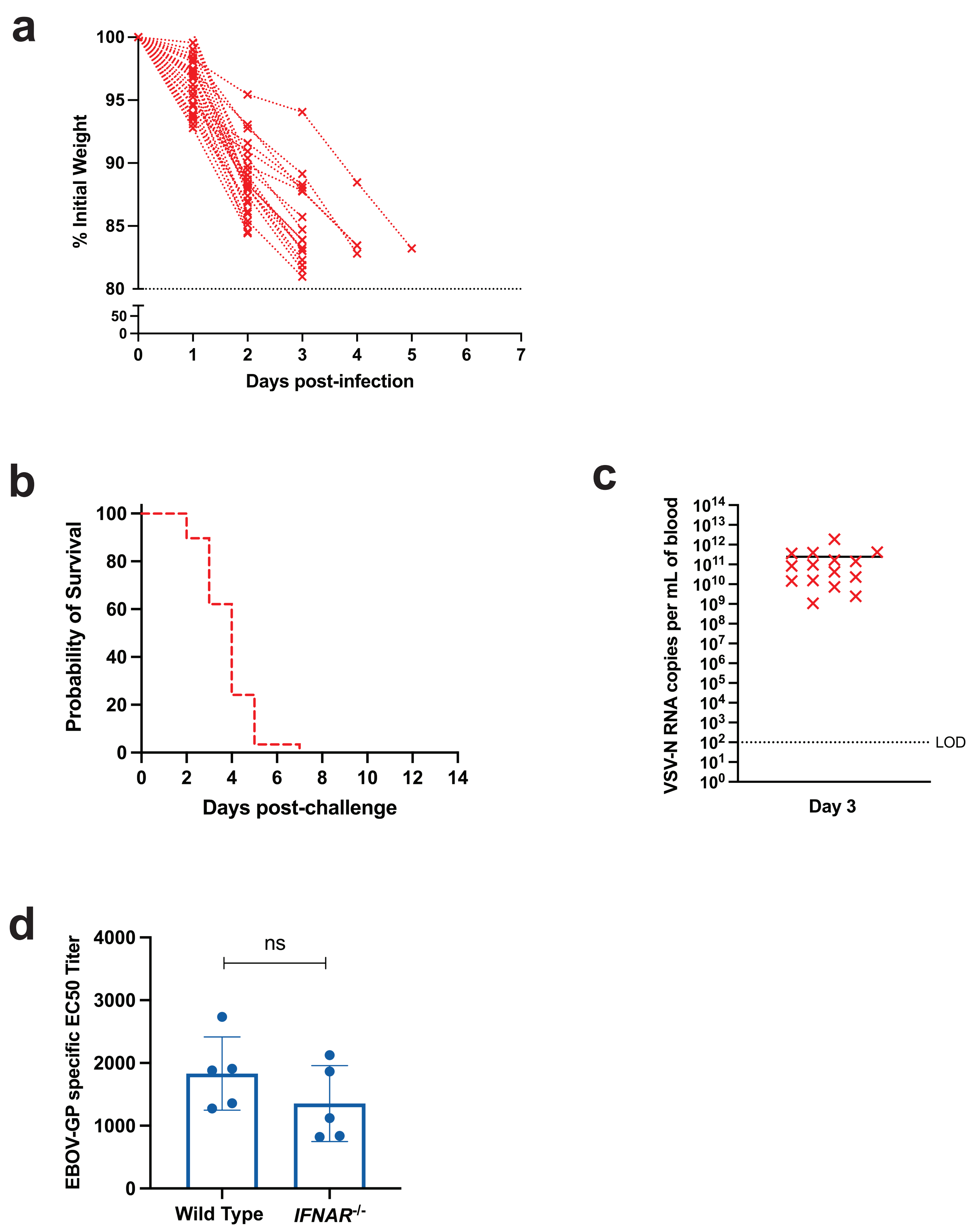

Supplementary Figure 4. Characterization of BSL3 surrogate challenge system for EBOV. **(a)** Weight curve of 30 unimmunized IFNAR-/- mice post-infection with a lethal dose of VSVΔG-EBOV-GP (5x105 PFU). **(b)** Survival curve of 30 unimmunized IFNAR-/- mice post-infection. **(c)** qPCR of VSV-N RNA copies per mL of blood at day 3 post-infection of 15 IFNAR-/- mice surviving to this time point. Limit of detection (LOD) of the assay is 100 copies per/mL (dotted line). **(d)** EBOV-GP specific ELISA in FILORAB1 and GLA-SE immunized wild type and IFNAR-/- 4 weeks post-immunization reported as average half-maximal effective concentration (EC50) titer (bars) determined from individual mouse serum (symbols) ELISA curves. Error bars represent SD from the mean. Statistics are by t-test of log-transformed EC50 titers. $p > 0.1234$ (ns), $p < 0.0332$ (*), $p < 0.0021$ (**), $p < 0.0002$ (***), $p < 0.0001$ (****).

Supplementary Figure 5

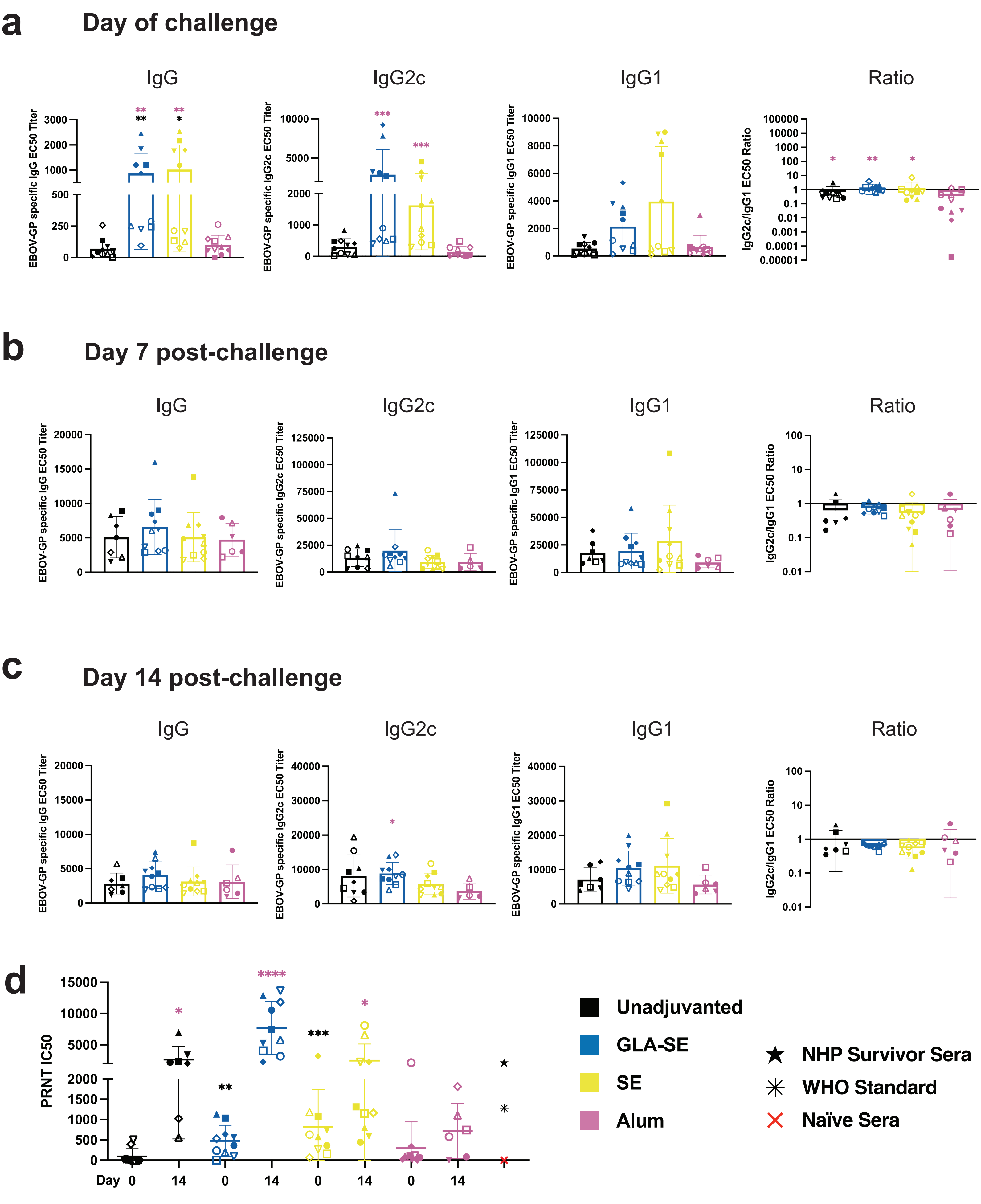

Supplementary Figure 5. EBOV-GP specific IgG responses before and after surrogate challenge. **(a)** EBOV-GP specific IgG, IgG2c, and IgG1 EC50 titers and isotype subclass ratio on the day of challenge and **(b)** day 7 and **(c)** day 14 post-challenge reported as average half-maximal effective concentration (EC50) titer (bars) determined from individual mouse serum (symbols) ELISA curves. **(d)** Neutralizing antibody titers on the day of challenge (day 0) and day 14 post-challenge reported as half-maximal inhibitory concentration (IC50) of serum dilution. NHP survivor sera (black star) is pooled from a previous EBOV challenge experiment. The WHO standard (black asterisk) consists of convalescent plasma pool. Error bars represent SD from the mean. Statistics are by one-way ANOVA with post-hoc Tukey's test of log-transformed EC50 titers. $p > 0.1234$ (ns), $p < 0.0332$ (*), $p < 0.0021$ (**), $p < 0.0002$ (***), $p < 0.0001$ (****).

Supplementary Figure 6

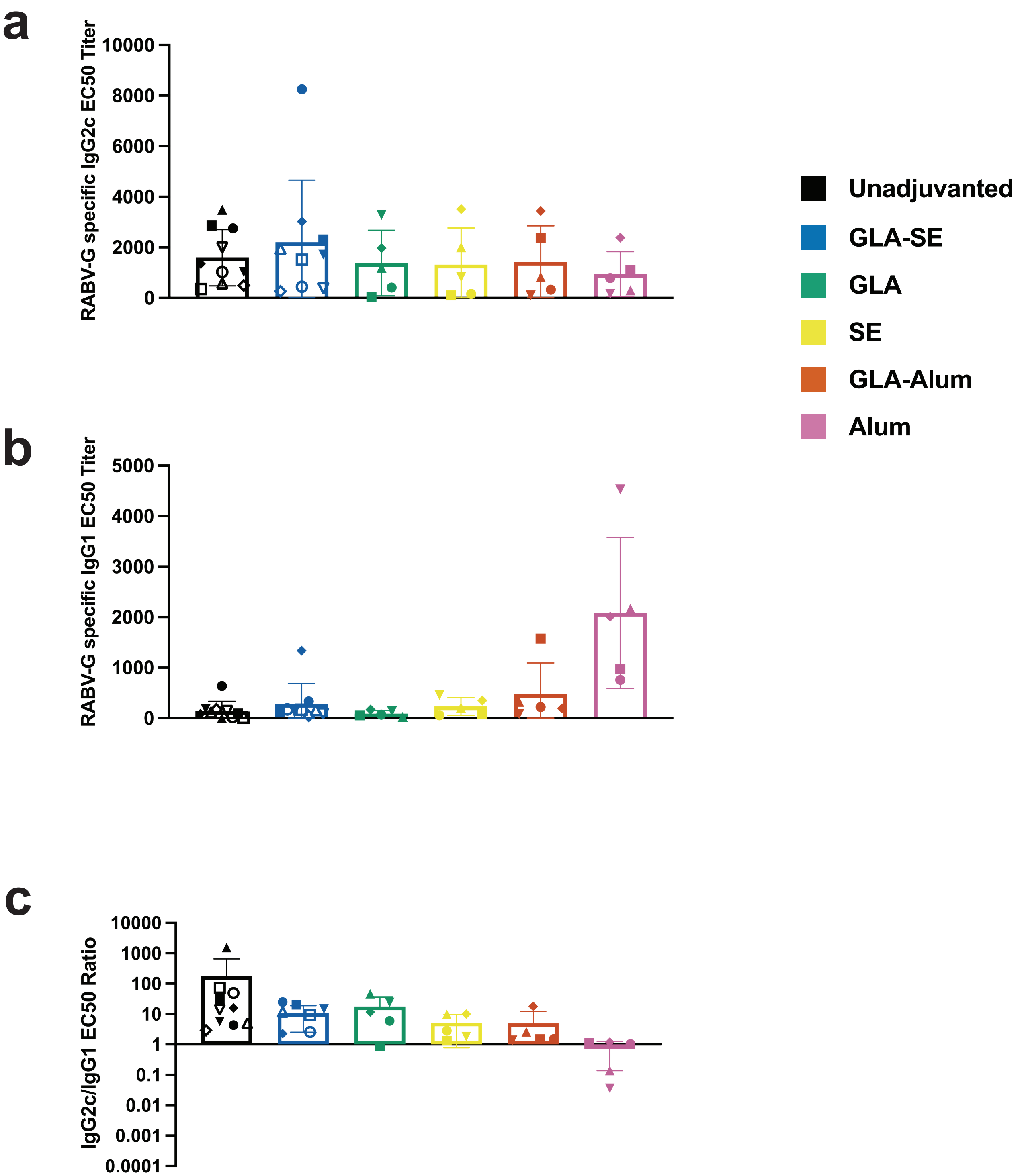

Supplementary Figure 6. RABV-G specific isotype subclass levels 1 year post-immunization. **(a)** RABV-G specific IgG2c, **(b)** IgG1, and **(c)** ratio of IgG2c/IgG1 titers at 1 year post-immunization reported as average half-maximal effective concentration (EC50) titer (bars) determined from individual mouse serum (symbols) ELISA curves. Error bars represent SD from the mean. Statistics are by one-way ANOVA with post-hoc Tukey's test of log-transformed EC50 titers. $p > 0.1234$ (ns), $p < 0.0332$ (*), $p < 0.0021$ (**), $p < 0.0002$ (***), $p < 0.0001$ (****).

Supplementary Figure 7

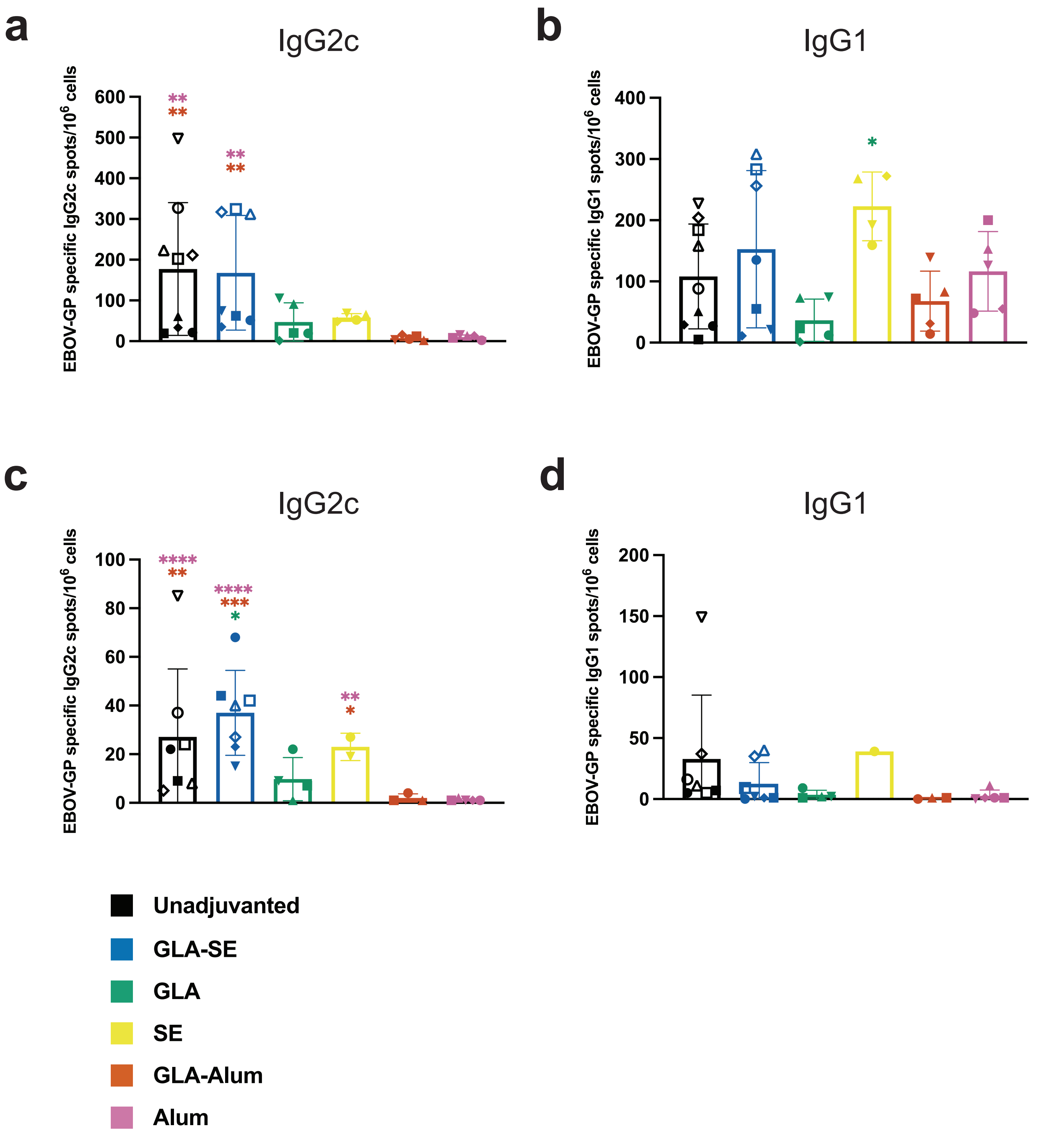

Supplementary Figure 7. EBOV-GP specific isotype subclass specificity of long-lived antibody-secreting cells (ASCs) in the bone marrow and spleen. **(a)** The average number (line) of EBOV-GP specific IgG2c and **(b)** IgG1 ASCs present in individual mouse bone marrow (symbols) at 1 year per adjuvant group. **(c)** The average number of EBOV-GP specific IgG2c and **(d)** IgG1 ASCs present in individual mouse spleens at 1 year per adjuvant group. Statistics are by one-way ANOVA with post-hoc Tukey's test of the log-transformed total number of cells or ratios. p > 0.1234 (ns), p < 0.0332 (*), p < 0.0021 (**), p < 0.0002 (***), p < 0.0001 (****).
